# A study on early diagnosis for fracture non-union prediction using deep learning and bone morphometric parameters

**DOI:** 10.3389/fmed.2025.1547588

**Published:** 2025-03-24

**Authors:** Hui Yu, Qiyue Mu, Zhi Wang, Yu Guo, Jing Zhao, Guangpu Wang, Qingsong Wang, Xianghong Meng, Xiaoman Dong, Shuo Wang, Jinglai Sun

**Affiliations:** ^1^State Key Laboratory of Advanced Medical Materials and Devices, Tianjin University School of Medicine, Tianjin, China; ^2^Department of Biomedical Engineering, Tianjin University School of Medicine, Tianjin, China; ^3^Radiology Department, Tianjin University Tianjin Hospital, Tianjin, China; ^4^Haihe Laboratory of Brain-Computer Interaction and Human-Machine Integration, Tianjin University School of Medicine, Tianjin, China

**Keywords:** micro-CT, fracture non-union prediction, bone morphometric parameters, deep learning, Mamba network

## Abstract

**Background:**

Early diagnosis of non-union fractures is vital for treatment planning, yet studies using bone morphometric parameters for this purpose are scarce. This study aims to create a fracture micro-CT image dataset, design a deep learning algorithm for fracture segmentation, and develop an early diagnosis model for fracture non-union.

**Methods:**

Using fracture animal models, micro-CT images from 12 rats at various healing stages (days 1, 7, 14, 21, 28, and 35) were analyzed. Fracture lesion frames were annotated to create a high-resolution dataset. We proposed the Vision Mamba Triplet Attention and Edge Feature Decoupling Module UNet (VM-TE-UNet) for fracture area segmentation. And we extracted bone morphometric parameters to establish an early diagnostic evaluation system for the non-union of fractures.

**Results:**

A dataset comprising 2,448 micro-CT images of the rat fracture lesions with fracture Region of Interest (ROI), bone callus and healing characteristics was established and used to train and test the proposed VM-TE-UNet which achieved a Dice Similarity Coefficient of 0.809, an improvement over the baseline's 0.765, and reduced the 95th Hausdorff Distance to 13.1. Through ablation studies, comparative experiments, and result analysis, the algorithm's effectiveness and superiority were validated. Significant differences (*p* < 0.05) were observed between the fracture and fracture non-union groups during the inflammatory and repair phases. Key indices, such as the average CT values of hematoma and cartilage tissues, BS/TS and BS/TV of mineralized cartilage, BS/TV of osteogenic tissue, and BV/TV of osteogenic tissue, align with clinical methods for diagnosing fracture non-union by assessing callus presence and local soft tissue swelling. On day 14, the early diagnosis model achieved an AUC of 0.995, demonstrating its ability to diagnose fracture non-union during the soft-callus phase.

**Conclusion:**

This study proposed the VM-TE-UNet for fracture areas segmentation, extracted micro-CT indices, and established an early diagnostic model for fracture non-union. We believe that the prediction model can effectively screen out samples of poor fracture rehabilitation caused by blood supply limitations in rats 14 days after fracture, rather than the widely accepted 35 or 40 days. This provides important reference for the clinical prediction of fracture non-union and early intervention treatment.

## 1 Introduction

Fractures are a global public health issue. The Global Burden of Disease Study 2019 (1) indicated that there were 178 million new fracture cases worldwide in 2019, with a total of 455 million cases. Fracture healing can be divided into four stages: the inflammatory phase, the repair phase, which include soft-callus formation phase and hard-callus formation phase, and the remodeling phase. Immediately after a fracture occurs, a hematoma forms (2). In the following weeks, the cartilage callus forms (3), which then undergoes mineralization a few weeks or months post-fracture (4), and finally, the original bone is remodeled over several months to years (5). However, among all fracture cases, ~5%−10% develop into non-union or delayed union, particularly in instances of compromised vascular supply (6), with the clinical diagnosis of non-union requiring 9 months (7, 8). Non-union of fractures is defined as a fracture that has not healed for at least 9 months and shows no signs of healing for 3 consecutive months (9). Delayed or non-union fractures can lead to long-term pain, disability, and repeated surgical interventions, consequently posing a significant economic burden and productivity loss to both the patient and society (10–12). Thus, early diagnosis of non-union fractures is crucial in terms of the formulation of treatment plans. Despite a plethora of research on the assessment of the progression of fracture healing (13–15), there is currently a lack of publicly available studies that directly utilize bone morphometric parameters for modeling the early diagnosis of non-union fractures.

The specific biological mechanisms behind non-union in avascular fractures remain unclear, and early radiological diagnostic characteristics and targets are not well-defined (13). Ethical and technical constraints have led to a lag in clinical prevention and early diagnosis of non-union, and existing imaging diagnostic methods like X-rays (14, 15) and Computed Tomography (CT) scans (16) often fail to capture the detailed features of fracture sites. The research on fracture healing is further hampered by the scarcity of high-quality public imaging datasets, which inhibits more in-depth studies. Consequently, the establishment of an imaging dataset that facilitates continuous observation of the healing process in ischemic fractures is crucial. Such a dataset would be invaluable for early diagnosis of non-union and for gaining a comprehensive understanding of the healing process.

Micro-Computed Tomography (micro-CT) technology, characterized by its high-resolution capabilities, has become an essential tool in non-invasive small animal imaging research (17). However, repeated scans throughout the healing process can be harmful to humans (18). Rats, with their strong vitality, exhibit minimal impact on healing processes due to weekly micro-CT imaging (19). Rats typically confirm non-union post-fracture in about 40 days, markedly shorter than the 270 days in humans, making them a suitable model for medical research (20). Therefore, this study chose rats as the experimental subjects, utilizing the research team's previous studies on animal models (21) and employing micro-CT imaging as the primary observation tool.

In recent years, with the rapid development of deep learning, models based on Convolutional Neural Networks (CNNs), such as U-Net (22), have made achieved significant progress in medical image processing (23). However, these models struggle to utilize global context effectively in complex 2D medical images (24). The incorporation of Transformer (25), including Swin Transformer (26) and Vision Transformer (ViT) (27), has improved segmentation accuracy but faces challenges with small medical image datasets, which are prone to overfitting. Recently, the Mamba model-based segmentation algorithms have drawn interest in medical imaging. Ruan and Xiang (28) introduced Vision Mamba UNet (VM-UNet), which combines Mamba blocks with the U-Net structure to enhance the model's ability to capture long-range dependencies, replacing traditional convolutional layers. This is particularly relevant for tasks like fracture area segmentation that involve dynamic changes, where there is a lack of specialized automated segmentation algorithms.

The aim of this study is to develop a high-resolution CT image dataset, identify the most indicative parameters for fracture non-union, and construct a predictive model for early diagnosis of non-union. To achieve the aforementioned goals, our main contributions are as follows:

(i) Based on our fracture animal models (21), a total of 45,275 micro-CT images were obtained from 12 rats across various stages of fracture healing (days 1, 7, 14, 21, 28, and 35 post-fracture). Among these, 2,448 frames containing the fracture lesion were annotated, including fracture Region of Interest (ROI), bone callus and healing characteristics, and a high-resolution fracture CT image dataset was established which can not only be used on fracture area segmentation and early diagnostic models for fracture non-union, but also for the study of other fracture-related algorithms and applications in medical education.

(ii) Based on the recently prominent high-performance semantic segmentation algorithm—VM-UNet (28), we have made adaptive improvements for the task of fracture area edge segmentation. Consequently, we proposed the Vision Mamba Triplet Attention and Edge Feature Decoupling Module UNet (VM-TE-UNet), which includes the Edge Feature Decoupling Module (EFDM) and Vision State Space Triplet Attention (VSS-TA) blocks, and also improved the loss functions.

(iii) The fracture site was quantitatively analyzed by extracting bone morphometric parameters based on data derived from micro-CT images, enabling a detailed assessment of the structural characteristics. Four micro-CT indicators were extracted and screened to characterize key changes in inflammatory hematoma tissue, cartilage tissue, mineralized cartilage tissue, and osteogenic tissue, including average CT value, Bone Area Fraction (BS/TS), Bone Volume Fraction (BV/TV), and Bone Area to Volume Ratio (BS/TV). On this basis, and taking into account the existing clinical diagnostic experience, we have constructed an early diagnosis model for non-union fractures. On the 14th day, the Area Under the Curve (AUC) (29), Accuracy (30), and Precision (31) of the early diagnosis model reached 0.995, 0.996, and 0.994, respectively, achieving the diagnosis of fracture non-union during soft-callus formation phase.

## 2 Material and methods

### 2.1 Research technical architecture

**Figure 1:**
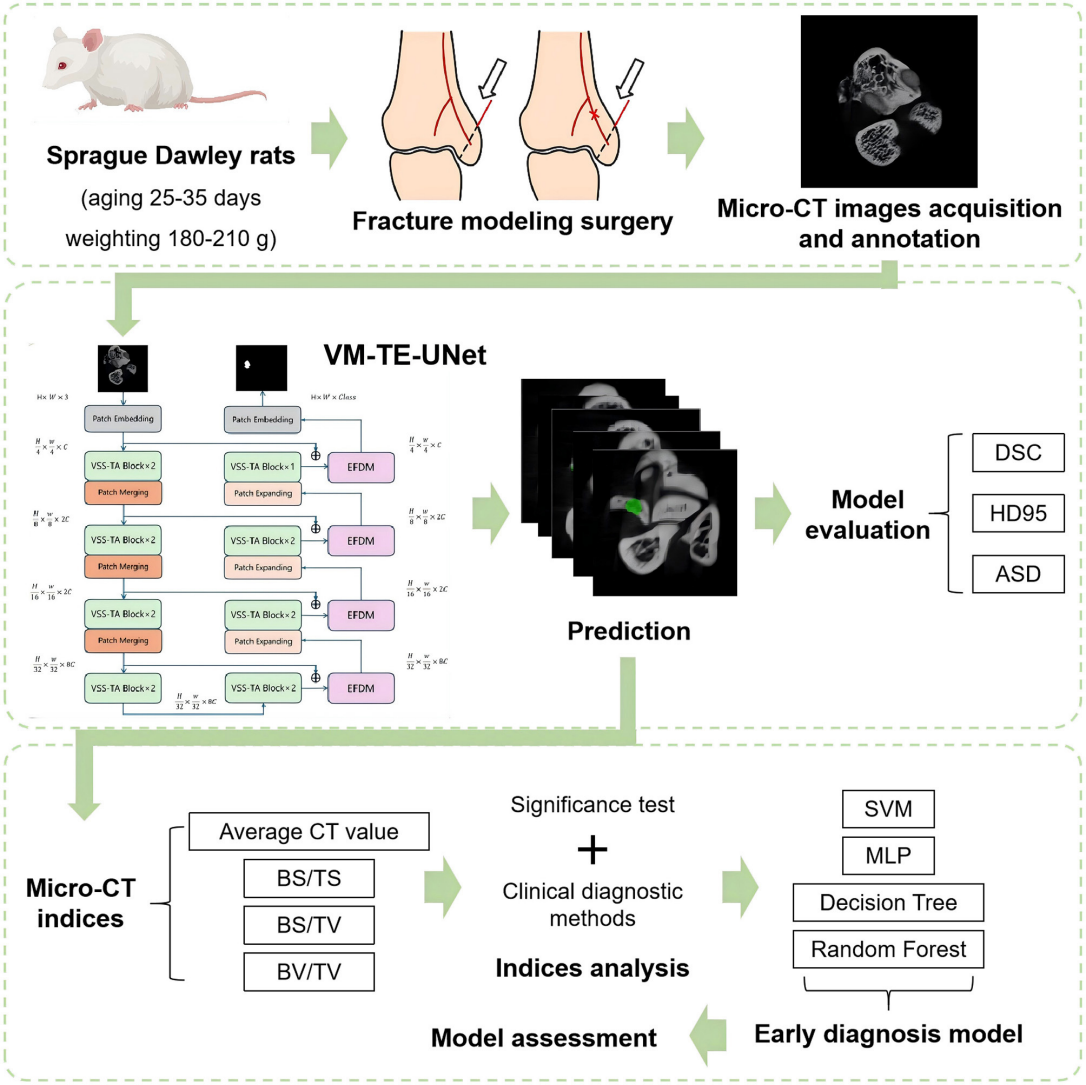
Overview of the technical roadmap.

Figure 1 illustrates the technical roadmap of this study. Through fracture modeling experiments, micro-CT images were obtained from 12 SD rats at days 1, 7, 14, 21, 28, and 35 post-fracture, with the frames containing the fracture area being annotated to establish a dataset. To address the issue of insufficient image segmentation accuracy in the fracture area, this study proposed VM-TE-UNet and conducted comparative and ablation experiments to verify the effectiveness and advantages of the algorithm. Four micro-CT indicators (the average CT value, BS/TS, BV/TV, and BS/TV) were extracted and screened to characterize key changes in inflammatory hematoma tissue, cartilage tissue, mineralized cartilage tissue, and osteogenic tissue. Based on this, and considering existing clinical diagnostic experience, we have constructed an early diagnosis model for non-union fractures.

### 2.2 Animal model and micro-CT dataset

#### 2.2.1 Experimental animals and surgical procedures

All animal experiments have been approved by the experimental animal ethics committee of Tianjin University of China (TJUE-2021-137) and were conducted in strict accordance with relevant regulations. The experiment used a total of 12 male Sprague Dawley (SD) rats (aging 25–35 days, weighting 180–210 g), which were randomly divided into two groups: the Fracture group (F group) and the Fracture + Saphenous Artery Cut-off group (F+S group), with 6 rats in each group. All rats were housed in the experimental animal center of the Fourth Central Hospital of Tianjin, China.

In the surgical procedures, first, the 12 rats were intraperitoneally injected with 0.3% pentobarbital sodium anesthetic at a ratio of 40–50 mg/kg. Once their breathing stabilized, the surface of the right medial malleolus was shaved, and the skin was incised along the long axis. Then, using a molding instrument developed by the research team, a 1 mm wide incision was created from the distal medial side of the ankle joint to the central medial malleolus, reaching part of the cortical bone and trabeculae without causing bone fragments to fall off. For the F+S group, the saphenous artery was cut off. Finally, the skin of both groups was sutured and the wounds were disinfected postoperatively.

#### 2.2.2 Micro-CT dataset

Within 35 days post-fracture at six time points (day 1, 7, 14, 21, 28, and 35), a total of 12 rats were scanned (SkyScan-1276, Bruker, Berlin, Germany), with an average of ~650 micro-CT images obtained per scan. The scans used isotropic voxel sizes of 10 μm, an exposure time of 486 ms, a tube voltage of 85 kV, and a tube current of 200 μA. The eligible experimental samples were selected from imaging workstations by two experienced radiologists based on stringent inclusion criteria, which included complete data, absence of significant artifacts or noise, appropriate grayscale levels, and clear tissue differentiation. High-quality, lossless TIFF data meeting these criteria were exported for further image processing and analysis.

Two sets of manual segmentations for all fracture lesions were performed independently by two different experienced radiologists. Dice Similarity Coefficient (DSC) (32) was used to assess the agreement between the two annotators on classifying whether a pixel from a Micro-CT image belonged to the fracture lesion or non-fracture lesion class.

The DSC is defined as:


(1) $$\begin{array}{l} DSC=\frac{2\times |A\cap B|}{\left|A|+|B\right|} \end{array}$$

where *A* and *B* are the annotated ROI of the two physicians.

When the DSC exceeds 0.95, it indicates a high level of consistency, and the annotation from either doctor can be selected as the segmentation ROI. If the DSC is below 0.95, the two doctors will discuss and reach a consensus to redefine a unified ROI.

### 2.3 Fracture lesion segmentation

#### 2.3.1 VM-TE-UNet architecture

**Figure 2:**
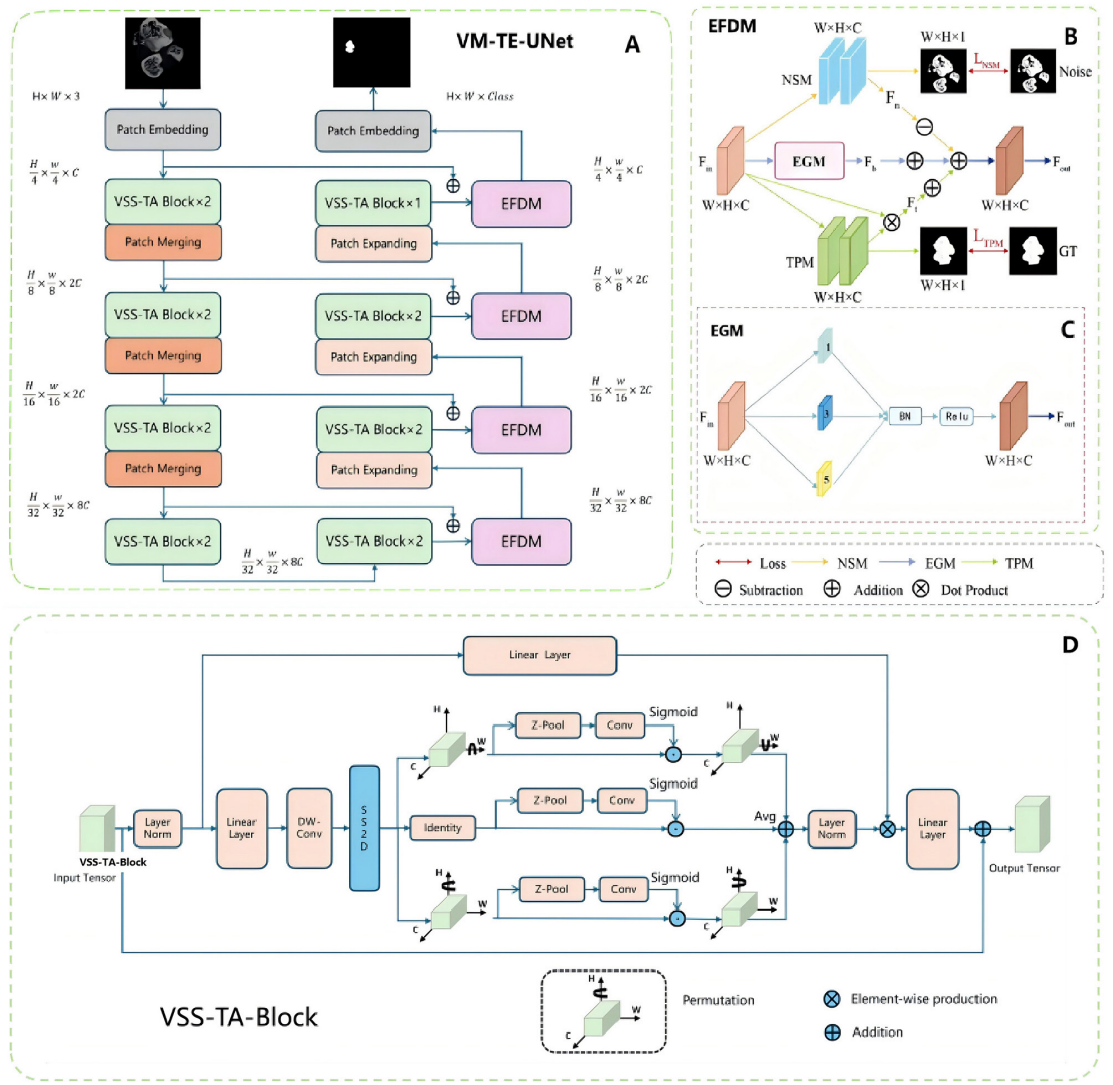
Overview of the deep learning model. **(A)** EFDM and VSS-TA block are applied to the overall network structure of VM-UNet. **(B)** In the specific implementation of EFDM, each rectangular box of the NSM module and TPM module represents a convolution layer, normalization layer and ReLU. **(C)** In the module structure diagram of the EGM, the numbers in each rectangular box represent the corresponding spatial convolution rate. **(D)** VSS-TA block, the improved attention mechanism network structure.

In this study, we proposed the VM-TE-UNet model (Figure 2A) for medical image segmentation, which is an adaption of the recently prominent high-performance semantic segmentation algorithm—VM-UNet (28). This novel model is designed to enhance segmentation performance through the integration of cutting-edge modules.

The network primarily consists of an encoder, a decoder, and the Edge Feature Decoupling Module (EFDM, Figure 2B). The EFDM is further divided into the Noise Suppression Module (NSM), the Texture Preservation Module (TPM), and the Edge-Guided Module (EGM, Figure 2C). The EGM enhances edge features through a series of atrous convolutions, batch normalization, and activation functions, thereby improving segmentation performance. The NSM learns the characteristics of noise by minimizing the difference between the predicted noise feature map and the ground truth noise mask, achieving noise suppression. The TPM learns and retains texture information in the fracture area by minimizing the difference between the predicted texture feature map and the ground truth texture mask.

The encoder consists of multiple Vision State Space Triplet Attention (VSS-TA) blocks (Figure 2D), each block followed by a patch merging operation, reducing spatial dimensions and increasing feature depth. The VSS-TA block is an integration of the Triplet Attention mechanism (33) on the basis of the original Vision State Space (VSS) block (34), aiming to strengthen the capability of feature extraction. The decoder recovers the spatial dimensions through the patch expanding operation and integrates the EFDM blocks after each hidden layer, thereby bolstering the segmentation accuracy.

Through this modular design, VM-TE-UNet can effectively capture and handle complex fracture area characteristics, significantly improving the accuracy and robustness of segmentation. The introduction of the Triplet Attention mechanism enhances the ability to focus on important features, while the EFDM block further optimizes the segmentation process through feature decoupling, ensuring accurate detection of fracture area boundaries and effective suppression of noise.

#### 2.3.2 Loss function

The VM-TE-UNet network was trained to minimize the total loss that consists of four parts: loss functions for the three branches of EFDM and the loss function for the VM-TE-UNet network. The total loss is calculated as:


(2) $$\begin{array}{l} L_{Total}=\overset{M}{\underset{j=1}{\sum }}L_{NSM}^{j}+\overset{M}{\underset{j=1}{\sum }}L_{TPM}^{j}+\overset{M}{\underset{j=1}{\sum }}L_{EGM}^{j}+\delta L_{SEG} \end{array}$$

where the *L*_*NSM*_, *L*_*TPM*_, and *L*_*EGM*_ are the loss functions for NSM, TPM, and EGM, *L*_*SEG*_ represents the segmentation loss of the VM-TE-UNet network, and δ is used to balance the weights of the different loss functions.

*L*_*EGM*_ is composed of weighted dice loss *L*_*Dice*_ and focal loss *L*_*Focal*_, which can be written as:


(3) $$\begin{array}{l} L_{EGM}=\alpha L_{Focal}+\left(1-\alpha \right)L_{Dice} \end{array}$$

where α is the weight factor.

*L*_*NSM*_ is defined as:


(4) $$\begin{array}{l} L_{NSM}=∥Y_{n}-M_{n}{∥}_{1} \end{array}$$

where *Y*_*n*_ is the predicted noise feature map and *M*_*n*_ is the ground truth noise mask.

*L*_*TPM*_ is defined as:


(5) $$\begin{array}{l} L_{TPM}=∥Y_{t}-M_{t}{∥}_{1} \end{array}$$

where *Y*_*t*_ is the predicted texture feature map and *M*_*t*_ is the ground truth texture mask.

*L*_*SEG*_ is composed of binary cross-entropy loss *L*_*Bce*_ and dice loss *L*_*Dice*_, which can be written as:


(6) $$\begin{array}{l} L_{SEG}=\lambda_{1}L_{Bce}+\lambda_{2}L_{Dice} \end{array}$$

where the weight factors of λ_1_ and λ_2_ are initially set to 1.

#### 2.3.3 Implementation details

In our experiments, the proposed VM-TE-UNet network is trained with Adam optimizer with a learning rate of 1e−5 and a batch size of 16. All the models are built using PyTorch platform and trained on a NVIDIA RTX 3090 GPU with a memory of 24 GB.

#### 2.3.4 Evaluation metrics

We employed three metrics to evaluate the performance of our model, which are as follows:

(i) The DSC is defined as the Equation 1, where *A* and *B* are the manual and predicted segmented masks, respectively. It is a statistical tool for comparing the similarity and consistency of sample sets.

(ii) The Hausdorff Distance (HD) (35) is the greatest of all the distances from a point in one set to the closest point in the other set, measuring the accuracy of boundary segmentation. It's defined as:


(7) $$\begin{array}{l} h\left(A,B\right)=\underset{a\in B}{\max}\underset{b\in B}{\min}d\left(a,b\right) \end{array}$$

(8) $$\begin{array}{l} HD\left(A,B\right)=\max\left(h\left(A,B\right),h\left(B,A\right)\right) \end{array}$$

where A and B are the two sample sets and *d (a, b*) is the Euclidean distance between A and B. The 95th Hausdorff Distance (HD95) mitigates the impact of outliers by calculating the distance at the 95th percentile, making it more suitable for measuring boundary differences in medical imaging.

### 2.4 Early diagnosis of avascular fracture non-union

#### 2.4.1 Indices extraction

The following indices of inflammatory hematoma tissue, cartilage tissue, mineralized cartilage tissue, and osteogenic tissue structure and composition were evaluated from the micro-CT images: the average CT value, BS/TS, BV/TV, and BS/TV (36). The segmentation of tissues within the fracture lesion was performed using specific Hounsfield Unit (HU) thresholds: inflammatory hematoma tissue was delineated at 0–224 HU, cartilage tissue at 225–330 HU, mineralized cartilage tissue at 331–700 HU, and osteogenic tissue at 701–1,000 HU (37).

The bone morphometric parameters were analyzed in this study to evaluate the structural characteristics of tissues, with all measurements conducted using CTvox (https://www.microphotonics.com/micro-ct-systems/visualization-software/) to ensure precision and consistency. The average CT value of a specific tissue was determined by averaging the CT values of all pixels within that tissue. For the four segmented tissues, the pixel count in each tissue was calculated for every image, yielding the Bone Area (BS). By summing the BS of all tissues, the Total Area (TS) was obtained. Similarly, micro-CT images of each sample were analyzed to calculate the Bone Volume (BV) and Total Volume (TV). These parameters were further used to derive ratios such as BS/TS, BV/TV, and BS/TV for each tissue, providing a comprehensive assessment of their structural properties.

#### 2.4.2 Early diagnosis model

Based on the extracted data, we performed statistical analyses and selected relevant indices to construct an early diagnostic model for fracture non-union by integrating clinical diagnostic approaches. This study employed a variety of machine learning classification methods to enhance the performance and robustness of the model, including Support Vector Machine (SVM) (38), Multilayer Perceptron (MLP) (39), Decision Tree (40), and Random Forest (41). The evaluation metrics include Accuracy (30), Precision (31) and F1 Score (42), which are defined as follows:


(9) $$\begin{array}{c} \{\begin{array}{c} Accuracy=\frac{TP+TN}{TP+TN+FP+FN}\\ Precision=\frac{TP}{TP+FP}\\ F1=\frac{Precision\times Recall}{Precision+Recall}\times 2 \end{array}\; \end{array}$$

where TP is true positives, TN is true negatives, FP is false positives, and FN is false negatives (43).

Additionally, the AUC (29), which stands for the area under the Receiver Operating Characteristic (ROC) curve, is also used to evaluate the performance of the model. The five-fold cross-validation was adopted.

## 3 Results

### 3.1 Fracture non-union dataset

**Figure 3:**
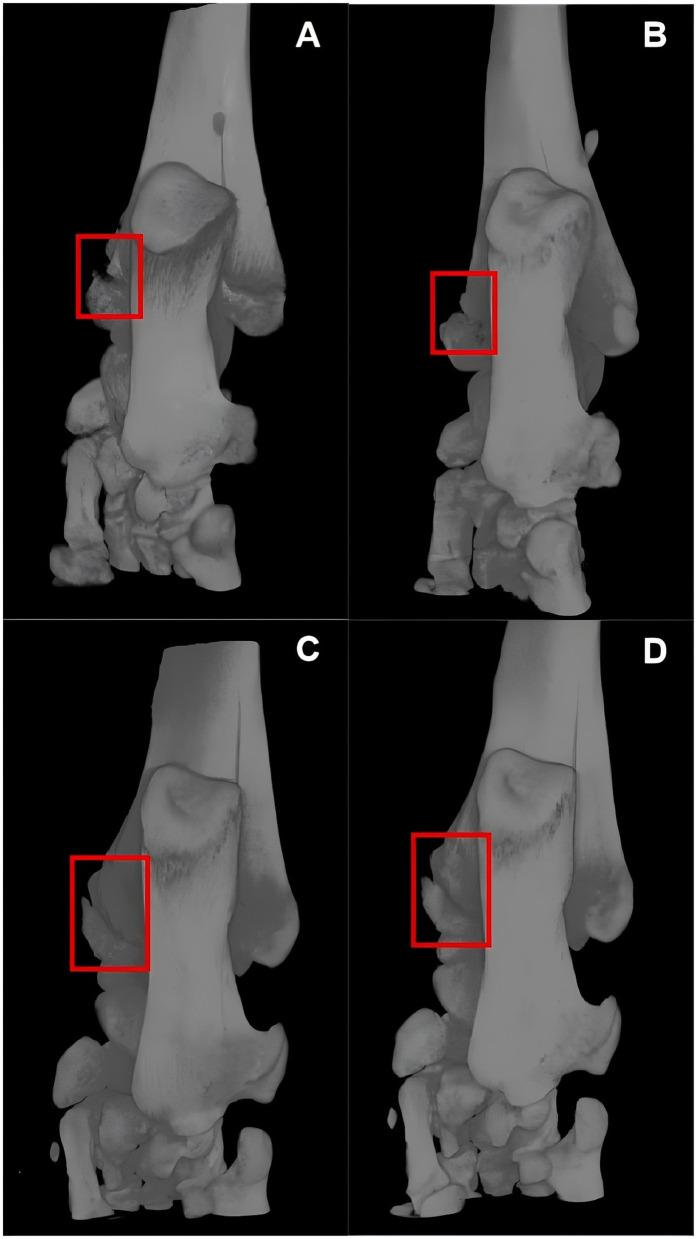
Micro-CT images of two rats in two groups. **(A, B)** Are micro-CT images of a group F rat on days 1 and 35 post-fracture. **(C, D)** Are micro-CT images of a group F+S rat on days 1 and 35 post-fracture.

Within 35 days after the fracture, at six time points (day 1, 7, 14, 21, 28, and 35), a total of 12 rats were scanned, collecting 45,275 micro-CT images, of which 2,448 frames contained the fracture lesion. Figure 3 shows the scan results of two rats in two groups. We made an incision in the medial malleolus of the rats (Figures 3A, C). After 5 weeks of healing, the medial malleolus of the group F rat finally healed at week 5 (Figure 3B), but there was no sign of healing in the F+S group (Figure 3D).

In the fracture segmentation part of this study, 9 rats were used as the segmentation training set, 1 rat as the validation set, and 2 rats as the test set. For the early fracture non-union prediction, all 12 rats were utilized in a five-fold cross-validation approach for machine learning, ensuring robust model evaluation and performance assessment.

### 3.2 Experimental results on fracture non-union dataset

#### 3.2.1 Comparison of different methods' segmentation results

The comparison of the proposed VM-TE-UNet with previous state-of-the-art methods on the avascular fracture non-union micro-CT dataset is presented in Table 1. In the domain of medical image segmentation, UNet (22) and Vision Transformer (ViT) (27) are established models, yet they exhibit suboptimal performance in this particular task. Specifically, ViT underperforms relative to UNet across all metrics, showing its limitations in segmenting fracture lesions. Conversely, Swin-UNet (44) and SegResNet (45) have demonstrated some performance improvements, albeit not markedly. EfficientNet (46) has achieved mixed results, with a DSC of 0.7313, indicating a marginal advancement in boundary treatment.

**Table 1:** The segmentation accuracy of different methods.

**Method**	**DSC**	**HD95**
UNet (22)	0.752 (0.702, 0.844)	18.7 (17.1, 20.3)
Vit (27)	0.694 (0.663, 0.751)	22.9 (22.3, 23.5)
Swin-UNet (44)	0.703 (0.652, 0.772)	24.6 (24.0, 25.2)
SegResNet (45)	0.729 (0.689, 0.778)	20.1 (19.2, 21.0)
EfficientNet (46)	0.731 (0.656, 0.828)	14.7 (14.0, 15.4)
VM-UNet (28)	0.765 (0.738, 0.882)	16.9 (15.1, 18.7)
nnUNet (52)	0.776 (0.696, 0.865)	15.8 (15.0, 16.6)
VM-TA-UNet (our)	0.798 (0.734, 0.876)	15.4 (13.4, 17.4)
VM-TE-UNet (our)	0.809 (0.752, 0.939)	13.1 (12.1, 14.1)

Ninety five percentage confidence interval is reported.

In comparison to the conventional UNet, VM-UNet (28) has substantially enhanced segmentation accuracy by incorporating the VSS block, achieving a DSC of 0.765. Furthermore, VM-TA-UNet, which introduces a Triplet Attention mechanism (33), has bolstered feature extraction capabilities, increasing the DSC to 0.798 and demonstrating superior performance in managing complex boundaries. Notably, the proposed VM-TE-UNet outperforms all other models across evaluation metrics, with a DSC and HD95 of 0.809, and 13.1, respectively. The experimental outcomes indicate that the proposed methodology effectively mitigates issues related to boundary noise and texture information interference.

**Figure 4:**
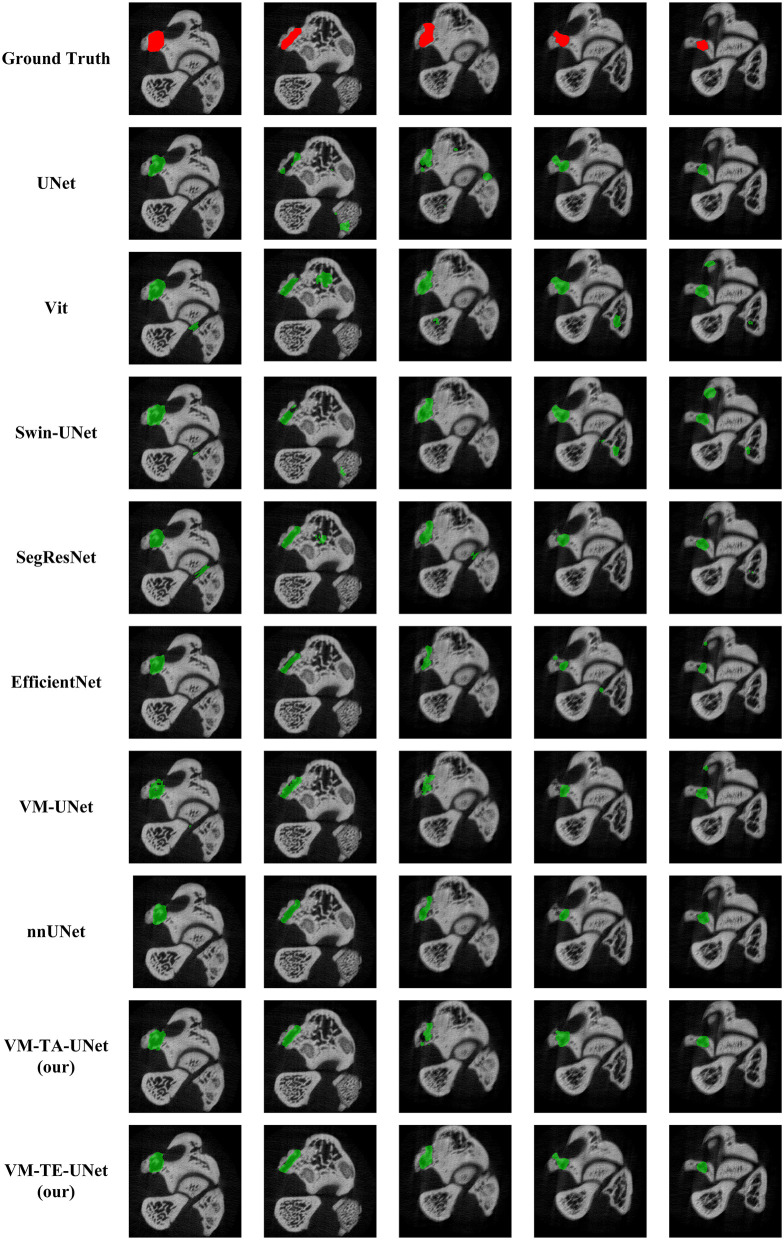
The segmentation results of different methods.

Figure 4 shows a more precise and fine segmentation output of the proposed network than the existing baselines. It is apparent that traditional UNet and ViT models exhibit subpar performance when segmenting complex boundaries, often resulting in notable false positive issues. Although Swin-UNet and SegResNet have marginally improved boundary segmentation and reduced some false positive occurrences, the segmentation results remain inaccurate due to the models' inability to fully capture the complex fracture boundaries.

In contrast, the VM-UNet model significantly enhances the recognition of complex boundary areas. However, due to the Mamba architecture's approach to image sequence processing, the model pays less attention to boundary information during segmentation, especially when dealing with irregular or blurred boundaries, leading to local information loss and boundary blurring. The VM-TA-UNet, which significantly improves segmentation performance, still experiences missegmentation in certain areas, particularly with more complex structures, indicating that despite the reduction in false positive phenomena, the model has not yet fully eliminated errors. Finally, the VM-TE-UNet, which integrates the EFDM, further enhances the model's segmentation capabilities. As can be seen from the figures, the segmentation results after EFDM improvement are smoother, with significantly enhanced boundary accuracy and effective control over false positive issues. Compared to other segmentation algorithms, VM-TE-UNet excels in processing noise information and capturing subtle boundary features, resulting in more precise and complete segmentation of fracture areas, providing more valuable technical support for the early diagnosis of fracture non-union.

#### 3.2.2 Attention improvement contrast experiment

Table 2 presents the performance of the different attention mechanisms in the fracture lesion segmentation task. A comparison was made among multiple spatial, channel, and hybrid attention mechanisms to evaluate their impact on segmentation accuracy (DSC and HD95). It is observed that single-type attention mechanisms, such as Non-local and SE, do not significantly enhance segmentation performance. In contrast, hybrid attention mechanisms like CBAM (47) and DANet (48) integrate feature attention across different dimensions, allowing for more accurate capture of complex structures in fracture areas. Notably, the Triplet Attention mechanism (33) demonstrates superior segmentation outcomes, achieving an encouraging DSC of 0.798 and significantly reducing HD95 to 15.4. By processing input tensors through rotations in height, width, and channel dimensions, Triplet Attention efficiently captures three-dimensional feature interactions, enhances local detail focus, improves global context understanding, and is computationally suitable for high-resolution medical imaging.

**Table 2:** Results of comparative experiments with different attention mechanisms.

**Attention mechanism category**	**Method**	**DSC**	**HD95**
Baseline model	VM-UNet	0.765 (0.738, 0.882)	16.9 (15.1, 18.7)
Spatial attention	+Non-local (53)	0.756 (0.708, 0.803)	21.3 (20.5, 22.7)
Spatial attention	+SK (54)	0.744 (0.663, 0.824)	12.1 (11.2, 13.6)
Channel attention	+SE (55)	0.716 (0.673, 0.759)	24.0 (23.1, 25.4)
Channel attention	+ECA (56)	0.722 (0.687, 0.757)	23.0 (22.3, 24.2)
Hybrid attention	+CBAM (47)	0.756 (0.676, 0.836)	21.3 (20.2, 23.0)
Hybrid attention	+DANet (48)	0.785 (0.725, 0.846)	15.7 (14.9, 16.8)
Hybrid attention	+AFF (57)	0.744 (0.673, 0.815)	20.6 (19.4, 22.3)
Hybrid attention (VM-TA-UNet)	Triplet attention (33)	0.798 (0.734, 0.876)	15.4 (13.4, 17.4)
Hybrid attention	+AxialAttention (58)	0.736 (0.703, 0.768)	24.6 (24.4, 24.7)

Ninety five percentage confidence interval is reported.

Enhancing VM-UNet's performance, these improvements also pave the way for integrating Mamba architecture with attention mechanisms, potentially increasing segmentation accuracy and fostering innovative, precise solutions in medical image segmentation.

#### 3.2.3 Ablation study

To verify the effectiveness of each branch within the EFDM, we conducted an ablation study. The results are shown in Table 3.

**Table 3:** Ablation study on the impact of EFDM.

**Method**	**EGM**	**NSM**	**TPM**	**DSC**	**HD95**
VM-TA-UNet	–	–	–	0.798 (0.734, 0.876)	15.4 (13.4, 17.4)
	√	–	–	0.804 (0.746, 0.862)	14.9 (14.1, 15.7)
	√	√	–	0.806 (0.724, 0.887)	14.4 (13.2, 15.6)
VM-TE-UNet	√	√	√	0.809 (0.752, 0.939)	13.1 (12.1, 14.1)

Ninety five percentage confidence interval is reported.

Compared to the basic VM-UNet without the introduction of any EFDM branches and with only the VSS block improved, the addition of the EGM branch alone increased the DSC to 0.804, and reduced HD95 to 14.9. Further inclusion of the NSM branch enhanced the DSC to 0.806, with HD95 lowering to 14.4. Ultimately, the incorporation of the TPM branch achieved the highest DSC of 0.809, with HD95 reducing to 13.1. The results indicate that EGM, NSM, and TPM branches, respectively, refine and enhance the segmentation results by strengthening boundary features, eliminating noise, and preserving texture information.

### 3.3 Early diagnosis of avascular fracture non-union

#### 3.3.1 Micro-CT indices

The average CT value, BS/TS, BS/TV, and BV/TV for both groups of rats are shown in Table 4.

**Table 4:** The average CT values, BS/TS, BS/TV, and BV/TV of the F group and F+S group on days 1, 7, 14, 21, 28, and 35 post-fracture.

**Group**	**Tissue**	**Day 1**	**Day 7**	**Day 14**	**Day 21**	**Day 28**	**Day 35**
**Average CT Value (HU)**							
F	Inflammatory hematoma	88.5 ± 30.2	**79.0** **±** **12.9**	**103** **±** **29.7**	**85.9** **±** **32.1**	**105** **±** **24.7**	92.8 ± 38.4
	Cartilage	**211** **±** **50.0**	**174** **±** **88.9**	**203** **±** **73.8**	**204** **±** **89.7**	**196** **±** **30.3**	203 ± 70.3
	Mineralized cartilage	**482** **±** **61.7**	**465** **±** **80.2**	491 ± 88.0	513 ± 78.9	**457** **±** **48.7**	**495** **±** **50.0**
	Osteogenic	**672** **±** **88.9**	**672** **±** **84.6**	698 ± 83.7	**720** **±** **58.2**	633 ± 64.3	**685** **±** **75.1**
F+S	Inflammatory hematoma	76.0 ± 38.0	**59.2** **±** **35.4**	**54.0** **±** **21.6**	**64.8** **±** **38.4**	**71.3** **±** **18.6**	82.5 ± 33.6
	Cartilage	**155** **±** **47.5**	**149** **±** **109**	**144** **±** **129**	**145** **±** **126**	**156** **±** **87.6**	198 ± 85.0
	Mineralized cartilage	**383** **±** **72.5**	**448** **±** **123**	482 ± 126	500 ± 88.9	**435** **±** **94.9**	**534** **±** **45.7**
	Osteogenic	**564** **±** **90.6**	**642** **±** **129**	681 ± 120	**685** **±** **89.7**	620 ± 103	**737** **±** **55.2**
**BS/TS (%)**							
F	Inflammatory hematoma	**4.83** **±** **5.58**	**5.54** **±** **4.56**	**3.97** **±** **5.00**	**2.31** **±** **2.55**	**6.89** **±** **4.80**	**3.66** **±** **2.91**
	Cartilage	**5.27** **±** **3.71**	**5.26** **±** **3.11**	**4.16** **±** **3.23**	**3.14** **±** **2.11**	**6.25** **±** **2.38**	**4.33** **±** **2.30**
	Mineralized cartilage	35.0 ± 7.81	32.4 ± 8.45	32.1 ± 6.76	**30.4** **±** **7.81**	**37.2** **±** **4.29**	**33.2** **±** **7.78**
	Osteogenic	**54.9** **±** **15.1**	**56.8** **±** **14.2**	**59.8** **±** **12.7**	**64.2** **±** **11.0**	49.6 ± 9.19	**58.8** **±** **11.9**
F+S	Inflammatory hematoma	**13.0** **±** **8.07**	**8.35** **±** **9.4**	**5.29** **±** **5.40**	**4.13** **±** **3.36**	**10.5** **±** **7.35**	**1.47** **±** **1.55**
	Cartilage	**8.94** **±** **3.59**	**6.11** **±** **4.34**	5.74 ± 4.72	**5.00** **±** **3.35**	**7.46** **±** **3.79**	**2.64** **±** **2.07**
	Mineralized cartilage	36.2 ± 5.73	32.9 ± 10.1	32.9 ± 13.5	**35.4** **±** **11.2**	**33.9** **±** **7.37**	**30.8** **±** **8.01**
	Osteogenic	**41.8** **±** **11.1**	**52.6** **±** **18.8**	56.1 ± 21.7	**55.5** **±** **16.7**	48.1 ± 15.5	**65.1** **±** **10.8**
**BS/TV (1/mm)**							
F	Inflammatory hematoma	**0.0661** **±** **0.107**	**0.0552** **±** **0.0627**	0.0646 ± 0.103	0.0333 ± 0.0906	**0.0689** **±** **0.0599**	**0.0460** **±** **0.0537**
	Cartilage	**0.0612** **±** **0.0879**	0.0532 ± 0.0555	0.0608 ± 0.0734	0.0412 ± 0.0851	0.0593 ± 0.0388	**0.0509** **±** **0.0491**
	Mineralized cartilage	**0.300** **±** **0.325**	0.313 ± 0.257	0.381 ± 0.292	**0.344** **±** **0.335**	**0.346** **±** **0.187**	**0.346** **±** **0.274**
	Osteogenic	**0.336** **±** **0.237**	**0.490** **±** **0.400**	**0.598** **±** **0.367**	**0.674** **±** **0.574**	**0.439** **±** **0.232**	**0.519** **±** **0.333**
F+S	Inflammatory hematoma	**0.182** **±** **0.128**	**0.0763** **±** **0.0835**	0.0655 ± 0.0734	0.0343 ± 0.0336	**0.0985** **±** **0.089**	**0.0146** **±** **0.0247**
	Cartilage	**0.139** **±** **0.0957**	0.0619 ± 0.0631	0.0710 ± 0.0683	0.0419 ± 0.0369	0.0704 ± 0.0674	**0.0241** **±** **0.0359**
	Mineralized cartilage	**0.570** **±** **0.336**	0.328 ± 0.321	0.355 ± 0.270	**0.266** **±** **0.192**	**0.290** **±** **0.260**	**0.224** **±** **0.242**
	Osteogenic	**0.648** **±** **0.367**	**0.400** **±** **0.262**	**0.410** **±** **0.240**	**0.311** **±** **0.203**	**0.310** **±** **0.195**	**0.370** **±** **0.243**
**BV/TV (%)**							
F	Inflammatory hematoma	8.66 ± 4.23	6.05 ± 2.14	5.85 ± 3.73	3.04 ± 1.14	7.55 ± 1.95	4.79 ± 2.40
	Cartilage	8.01 ± 2.24	5.83 ± 1.55	5.50 ± 1.60	3.77 ± 0.843	6.50 ± 0.755	5.30 ± 1.30
	Mineralized cartilage	39.3 ± 5.90	34.3 ± 5.51	34.5 ± 0.41	31.5 ± 3.35	37.9 ± 0.83	36.0 ± 4.52
	Osteogenic	44.0 ± 8.36	53.8 ± 9.19	54.2 ± 4.92	**61.7** **±** **3.83**	48.1 ± 1.88	54.0 ± 8.22
F+S	Inflammatory hematoma	11.8 ± 3.32	8.80 8 4.00	7.27 ± 3.71	5.25 ± 3.50	12.8 ± 4.03	2.30 3 4.96
	Cartilage	9.00 ± 2.53	7.14 ± 1.14	7.88 ± 1.93	6.41 ± 1.27	9.15 ± 2.32	3.82 ± 1.45
	Mineralized cartilage	37.0 ± 3.87	37.9 ± 5.63	39.4 ± 1.01	40.7 ± 1.37	37.7 ± 2.41	35.4 ± 5.38
	Osteogenic	42.1 ± 9.72	46.2 ± 3.70	45.5 ± 3.64	**47.6** **±** **3.28**	40.3 ± 5.12	58.5 ± 5.00

Bold indicates p < 0.05 in comparison of the two groups in the same day. Mean and standard deviations are reported.

On day 1 postoperatively, the average CT value of the inflammatory hematoma tissue in the F group was 88.5 HU, and in the F+S group, it was 76.0 HU, with no significant difference. However, on days 7, 14, 21, and 28 post-fracture, the values in the F group (79.0, 103, 85.9, and 105 HU) were significantly higher than in the F+S group (59.2, 50.4, 64.8, and 71.3 HU). Similarly, the average CT value of the cartilage tissue in the F group (211, 174, 203, 204, and 196 HU) were significantly higher than in the F+S group (155, 149, 144, 145, and 156 HU) on days 1, 7, 14, 21, and 28 post-fracture.

On days 1, 7, 14, 21, and 28 post-fracture, the BS/TS of the inflammatory hematoma tissue and cartilage tissue in the F group (4.83%, 5.54%, 3.97%, 2.31%, and 6.89% for inflammatory hematoma tissue, respectively; 5.27%, 5.26%, 4.16%, 3.14%, and 6.25% for cartilage tissue, respectively) were significantly lower than in the F+S group (13.0%, 8.35%, 5.29%, 4.13%, and 10.5% for inflammatory hematoma tissue; respectively, 6.11%, 5.74%, 5.00%, and 7.46% for cartilage tissue, respectively). On day 21 post-fracture, the BS/TS of the mineralized cartilage tissue in the F group (30.4%) was lower than in the F+S group (35.4%). However, on days 28 and 35 post-fracture, the values in the F group (37.2% and 33.2%) were significantly higher than in the F+S group (33.9% and 30.8%).

On day 1 postoperatively, the BS/TV of the inflammatory hematoma tissue and cartilage tissue in the F group (0.0661 and 0.0612 mm^−1^) were significantly higher than in the F+S group (0.182 and 0.139 mm^−1^). On day 1 postoperatively, the BS/TV of the mineralized cartilage tissue in the F group (0.300 mm^−1^) was lower than in the F+S group (0.570 mm^−1^). However, on days 21, 28, and 35 post-fracture, the values in the F group (0.344, 0.346, and 0.346 mm^−1^) were significantly higher than in the F+S group (0.266, 0.290, and 0.224 mm^−1^). The BS/TV of the osteogenic tissue in the F group (0.336 mm^−1^) was lower than in the F+S group (0.648 mm^−1^) on day 1 postoperatively. However, the values in the F group (0.490, 0.598, 0.674, 0.439, and 0.519 mm^−1^) were significantly higher than in the F+S group (0.400, 0.410, 0.311, 0.310, and 0.370 mm^−1^) on days 7, 14, 21, 28, and 35 post-fracture.

On day 21 post-fracture, the BV/TV of the osteogenic tissue in the F group was 61.7%, while in the F+S group, it was 47.6%, with a significant difference.

#### 3.3.2 Early diagnosis model

According to the analysis presented in Section 3.3.1, we selected the following 10 indices for constructing the early diagnosis model of fracture non-union: (i) the average CT value of inflammatory hematoma and cartilage tissues, (ii) the BS/TS for all four types of tissues, (iii) the BS/TV of mineralized cartilage and osteogenic tissues, and (iv) the BV/TV of osteogenic tissue. Table 5 lists the AUC, Accuracy, Precision, and F1 Score of SVM, MLP, Decision Tree, and Random Forest models at different time points post-fracture.

**Table 5:** The performance of SVM, MLP, decision tree, and random forest models at different time points post-fracture.

**Model**	**Day**	**AUC**	**Accuracy**	**Precision**	**F1 Score**
SVM	7	0.917 (0.895, 0.940)	0.916 (0.893, 0.939)	0.922 (0.906, 0.937)	0.916 (0.893, 0.939)
	14	0.966 (0.937, 0.994)	0.962 (0.930, 0.993)	**0.987 (0.974, 1.001)**	0.970 (0.945, 0.994)
	21	0.797 (0.772, 0.822)	0.866 (0.842, 0.890)	0.886 (0.868, 0.905)	0.910 (0.889, 0.930)
	28	**0.970 (0.944, 0.996)**	**0.979 (0.962, 0.997)**	0.970 (0.946, 0.994)	**0.985 (0.972, 0.997)**
	35	0.814 (0.804, 0.824)	0.796 (0.773, 0.819)	0.896 (0.84, 0.952)	0.811 (0.788, 0.833)
MLP	7	0.971 (0.959, 0.982)	0.970 (0.957, 0.983)	0.967 (0.936, 0.997)	0.970 (0.954, 0.986)
	14	**0.995 (0.978, 1.012)**	**0.996 (0.980, 1.01)**	**0.994 (0.975, 1.01)**	**0.997 (0.98, 1.01)**
	21	0.934 (0.923, 0.944)	0.950 (0.94, 0.961)	0.967 (0.955, 0.979)	0.965 (0.957, 0.974)
	28	0.983 (0.963, 1.00)	0.988 (0.971, 1.01)	0.984 (0.964, 1.00)	0.992 (0.977, 1.01)
	35	0.901 (0.894, 0.908)	0.900 (0.894, 0.905)	0.928 (0.920, 0.935)	0.912 (0.908, 0.916)
Decision tree	7	0.950 (0.924, 0.977)	0.950 (0.922, 0.978)	0.959 (0.901, 1.02)	0.950 (0.916, 0.983)
	14	0.964 (0.952, 0.977)	0.973 (0.962, 0.985)	**0.979 (0.964, 0.994)**	0.980 (0.968, 0.992)
	21	0.935 (0.917, 0.954)	0.948 (0.938, 0.959)	0.971 (0.952, 0.989)	0.966 (0.956, 0.975)
	28	**0.964 (0.940, 0.989)**	**0.973 (0.959, 0.987)**	0.966 (0.955, 0.976)	**0.980 (0.972, 0.988)**
	35	0.840 (0.816, 0.865)	0.841 (0.825, 0.856)	0.867 (0.841, 0.894)	0.865 (0.852, 0.877)
Random forest	7	0.970 (0.962, 0.978)	0.970 (0.963, 0.977)	0.974 (0.974, 0.974)	0.970 (0.962, 0.978)
	14	0.980 (0.969, 0.992)	0.989 (0.977, 1.00)	**0.991 (0.971, 1.01)**	0.992 (0.982, 1.003)
	21	0.956 (0.929, 0.983)	0.959 (0.947, 0.971)	0.979 (0.961, 0.996)	0.972 (0.963, 0.98)
	28	**0.990 (0.969, 1.01)**	**0.991 (0.978, 1.00)**	0.990 (0.971, 1.01)	**0.993 (0.984, 1.00)**
	35	0.870 (0.858, 0.881)	0.870 (0.859, 0.880)	0.891 (0.88, 0.901)	0.890 (0.881, 0.898)

Bold indicates the best. Ninety five percentage confidence interval is reported.

On the first day post-fracture, due to inter-individual variations among rats, the micro-CT indices exhibited significant discrepancies, which impacted the early diagnostic model. Therefore, we excluded the data of the first day when establishing the model. The SVM, MLP, decision tree, and random forest models demonstrated optimal performance on days 28, 14, 28, and 28, respectively. Moreover, among all models and time points, the MLP model on day 14 post-fracture achieved the highest evaluation metrics, with AUC, Accuracy, Precision, and F1 Score reaching 0.995, 0.996, 0.994, and 0.997, respectively. In summary, the MLP model is capable of early diagnosis of fracture non-union on day 14 post-fracture in rats, which is the soft-callus formation phase and equivalent to a month post-fracture in humans.

These analyses indicate that our early diagnosis model has the potential to achieve the diagnosis during the soft-callus formation phase of fracture healing. Relative to the current diagnosis of non-union after 9 months post-fracture, the diagnosis was advanced by 8 months, thereby providing a golden time window for early intervention and treatment.

## 4 Discussion

In this study, we established a high-resolution fracture CT image dataset, proposed the VM-TE-UNet for fracture areas segmentation, extracted micro-CT indices, and established an early diagnostic model for fracture non-union, achieving the diagnosis of fracture non-union during soft-callus formation phase.

In this study, experiments were conducted based on our animal model (21), and a total of 12 rats' micro-CT imaging data at various stages of healing (on days 1, 7, 14, 21, 28, and 35 post-fracture) were collected, ultimately accumulating 2,448 high-resolution images of the rat ankle healing area. Subsequently, after preprocessing and annotation of the images, a micro-CT dataset was formed.

VM-TE-UNet, an advanced algorithm for segmenting rat ankle fracture areas, has been successfully developed. It builds on the VM-UNet, incorporating the Triple Attention mechanism into the VSS-TA block to boost feature capture. The VM-TA-UNet with this module achieved a DSC of 0.798, an improvement over the baseline's 0.765. To enhance performance, the EFDM was integrated into the final VM-TE-UNet model, which effectively separates boundary, noise, and texture from feature maps. Ablation studies showed that VM-TE-UNet with EFDM achieved a higher DSC of 0.809 and lowered the HD95 to 13.1, outperforming the model without EFDM and confirming EFDM's role in improving segmentation precision and minimizing false positives.

Currently, the clinical diagnosis of fracture non-union primarily relies on physical examination and radiographic evaluation. The physical examination includes assessing the presence or not of pain and restricted movement in the affected limb and its adjacent joints, which is aimed at confirming the blood supply to the distal part of the affected limb and determining the presence of pseudoarthrosis (49, 50). Localized soft tissue swelling that compresses blood vessels and fractures that damage blood vessels can both lead to poor blood supply. The poor blood may impair callus formation, therefore delay fracture healing.

Among imaging methods, X-ray is the basic method for diagnosing non-union. The scoring systems such as the Radiographic Union Score for Tibial Fractures (RUST) (14) and the Radiographic Union Score for Hip (RUSH) (51) can greatly improve the accuracy of non-union diagnosis. In the RUST system, the focus is on the presence of callus, fracture line, and bridging callus; whereas the RUSH system further scores cortical bridging, cortical disappearance, trabecular consolidation, and trabecular disappearance. The advantages of radiographic union scores are low cost, rapid, ease of implementation, suitability for use across multiple anatomic sites, and high diagnostic accuracy. However, they are semi-quantitative and subjective, which may lead to wide scoring discrepancies in the early diagnosis of non-union. Additionally, a significant drawback of X-rays is the inability to perform 3D imaging, with diagnostic accuracy being greatly influenced by imaging angles. In cases where X-ray film reveals no apparent non-union despite the presence of clinical symptoms, CT is employed for further diagnosis.

Rats reach the soft-callus formation phase 2–3 weeks after fracture, which is equivalent to 1 month after a human fracture. The average CT value can reflect the mineral density of various tissues.

On days 1, 7, 14, 21, and 28 post-fracture (the inflammatory phase and repair phase) the average CT values of the inflammatory hematoma and cartilage tissues in the F+S group are significantly lower than those in the F group, indicating that callus formation and mineralization are hindered in the F+S group. This is consistent with the theory that the soft-callus formation phase is prolonged and mineralization is affected under ischemic conditions in rats. The changes in the BS/TS of the mineralized cartilage tissue during the early hard-callus formation phase can reflect the negative effects of ischemia on cartilage callus mineralization, consistent with the theory. The BS/TV of osteogenic and mineralized cartilage tissue during the repair phase indicates that mineralization is hindered in the F+S group. The differences in BV/TV between the two groups during the hard-callus formation phase also support this conclusion. The differences in these indices correspond to the clinical method of diagnose the fracture non-union by assessing the presence of callus with X-rays.

The BS/TV of inflammatory hematoma and cartilage tissues in the inflammatory phase reflects the high proportion of soft tissue in the lesion area of the F+S group during this period. In the inflammatory phase and repair phase, the BS/TS of the inflammatory hematoma and cartilage tissues also indicates that the proportion of soft tissue in the lesion area is high in the non-union group of rats, corresponding to the clinical assessment of local soft tissue swelling causing vascular compression to diagnose non-union.

Based on the extracted micro-CT indices, a machine learning model for the early diagnosis of fracture non-union was constructed by comparing multiple models at multiple time points. The study revealed that on the 14th day, the MLP achieved an AUC of 0.995, an Accuracy of 0.996, and a Precision of 0.994, achieving the diagnosis of fracture non-union during soft-callus formation phase. The SVM, Decision Tree, and Random Forest models all exhibited optimal performance on the 28th day, achieving the diagnosis during hard-callus formation phase. The superior performance of the MLP in the study may be attributed to the dataset's complex non-linear boundaries, which the MLP, with its multi-layered structure and non-linear activations, can better capture, thereby enhancing classification performance. In contrast, SVM requires an appropriate kernel function to address non-linear issues, and Decision Trees and Random Forests may necessitate more sophisticated feature engineering to improve performance. Furthermore, the dataset comprised 2,448 samples, which may be a suitable quantity for the MLP. Meanwhile, SVM's computational complexity significantly increases with larger sample sizes. The input parameters consisted of 10 features, a moderate feature set for the MLP, which can effectively manage such a quantity. Decision Trees and Random Forests, on the other hand, may require more data to prevent overfitting.

It can be observed that early diagnostic models perform better on day 14 or 28 than on day 35 post-fracture, which contradicts common understanding. We speculate that there are two possible reasons for this phenomenon: on one hand, the limited sample size may lead to statistical errors, and on the other hand, there might be a muscular compensation mechanism that obscures the radiological features, thereby making the 14–28 days a more favorable time for diagnosing non-union fractures.

## 5 Conclusion

We believe that our prediction model based on micro-CT bone morphometric parameters can effectively screen out samples of poor fracture rehabilitation caused by blood supply limitations in rats 14 days after fracture, rather than the widely accepted 35 or 40 days. And we plan to further develop and validate the model proposed in this paper in human trails for clinical prediction for fracture non-union, providing a new approach for early intervention and treatment.

## Data Availability

The raw data supporting the conclusions of this article will be made available by the authors, without undue reservation.
